# Dual mobility for total hip arthroplasty revision surgery: A systematic review and metanalysis

**DOI:** 10.1051/sicotj/2021015

**Published:** 2021-03-22

**Authors:** Placella Giacomo, Bettinelli Giulia, Pace Valerio, Salini Vincenzo, Antinolfi Pierluigi

**Affiliations:** 1 Hospital San Raffaele – Orthopaedic Department Milan 20132 Italy; 2 Università Vita e Salute Milan 20132 Italy; 3 Università degli Studi di Perugia Perugia 06123 Italy

**Keywords:** Dual mobility, Revision, Dislocation, Hip prosthesis

## Abstract

*Introduction*: Revision THA (R-THA) is thought to have a higher complication rate if compared to primary THA. Dual Mobility (DM) implants have been designed aiming for achieving greater stability, with good clinical results. However, scarce material can be found about the real improvements provided by this type of implant compared to traditional implant in Revisions of Total Hip Arthroplasties. *Methods*: A systematic review and meta-analysis of comparative studies were performed in December 2019. This was in accordance with the guidelines of Preferred Reporting Items for Systematic reviews and Meta-Analyses (PRISMA). Our primary outcome measure was overall survivorship and dislocation rate, either treated with a conservative method or requiring surgery. *Results*: Regarding the overall implant survival, we found a slight significant risk ratio, with a statistically meaningful difference between the two groups in questions in favour of the DM implant. A statistically significant difference in favour of the DM group turned out considering only the Dislocation rate Risk ratio and the aseptic loosening risk as well. No statistical difference was found between the two groups about the risk ratio of infection. *Discussion*: A steady increase of evidence is demonstrating the efficacy of using a DM cup system in THA revisions with low dislocation rates, but currently there is no study in the literature that demonstrates with statistically significant evidence. The main finding of the present study is that implant’s Survivor and prevention of dislocation at medium follow-up showed better results with a DM if compared to a fixed-bearing cup, for Revision THA.

## Introduction

Revision THA (R-THA) is known to be a challenging type of surgery. This is thought to be caused by technical difficulties and higher complications rates in comparison with primary THA [1]. Re-revision THA risk at mid-follow-up has been recorded to be between 13% and 15.8% in the present literature [2, 3].

There are many factors able to cause the failure of R-THA: these have been found to be mostly instability and aseptic loosening. It has been reported that the dislocation rate in R-THA is almost 3 times higher than primary THA [4, 5].

It has been shown that complex and multifactorial factors may cause instability after R-THA: different leg length or inadequate abductor lever arm, poor quality of soft tissues and bone, prosthesis-bone impingement, and finally the one that seems to be the most significant: mal-positioning of the implants with regards to both acetabular side and femoral offset [6, 7].

Several surgical options have been studied for R-THA, but the use of Dual Mobility (DM) has some important advantages. DM provides the opportunity to improve implant stability giving the presence of a large Polyethylene liner at the level of the internal bearing. This larger liner works as a large femoral head and is able to produce a rise in jump distance. Moreover, DM does not cause increasing constraint at the level of the implant-bone interface but allows improvements with regards to the load dispersion interface [8, 9].

In the recent past, several authors described the most significant disadvantages of DM. These were thought to be: intra-prosthetic dislocation (IPD), aseptic loosening caused by polyethylene (PE) wear increment, and increased infection rate. These occurrences have been found to be less common in the newest generations of DMC and PE [10, 11].

For these reasons, DMC has recently attracted the interest of many surgeons, given the encouraging good overall results and the contemporary achievement of lower dislocation rates and very good clinical final results. However, the real efficacy of DM implants compared to fixed-bearing (FB) implants for R-THA is still associated to a relative lack of evidence due to the paucity of works on this matter.

The background aim of our research was to perform a systematic review and meta-analysis of comparative studies describing the use of DMC for R-THA. The final aim was to collect and sum up what is already known in this field in order to highlight the most current and up-to-date evidence presented in the literature to provide the highest level of evidence for each key aspect of this type of surgical option.

## Methods

A systematic review and meta-analysis of comparative studies was lead in December 2019. This was in conformity with the guidelines of Preferred Reporting Items for Systematic reviews and Meta-Analyses (PRISMA) [12].

Customized structured electronic searches were performed by two independent reviewers in PubMed, Google Scholar, Cochrane Library, and EMBASE using the following key terms: Revision AND tripolar OR double-mobility OR dual-mobility OR dual mobility OR double mobility OR hip OR total hip arthroplasty OR total hip replacement OR hip prosthesis.

We included all articles published from 2016 to December 2019.

Reference lists of eligible studies were also analyzed. Titles and abstracts were examined, and articles were identified for full-text review. Studies meeting inclusion criteria were evaluated in detail, and study characteristics were extracted using a standardized approach. Quantitative and qualitative characteristics of the studies were summarized. This was followed by analysis and synthesis. Inclusion and exclusion criteria were designed to capture studies on our selected key aspects of the use of DMC for R-THA.

The primary outcome measure was set as dislocation rate, either if it was treated with closed reduction or required surgical intervention. Secondary outcome measures of the review were: overall survival of implants, aseptic loosening, and infection rate.

### Eligibility criteria

**Figure 1:**
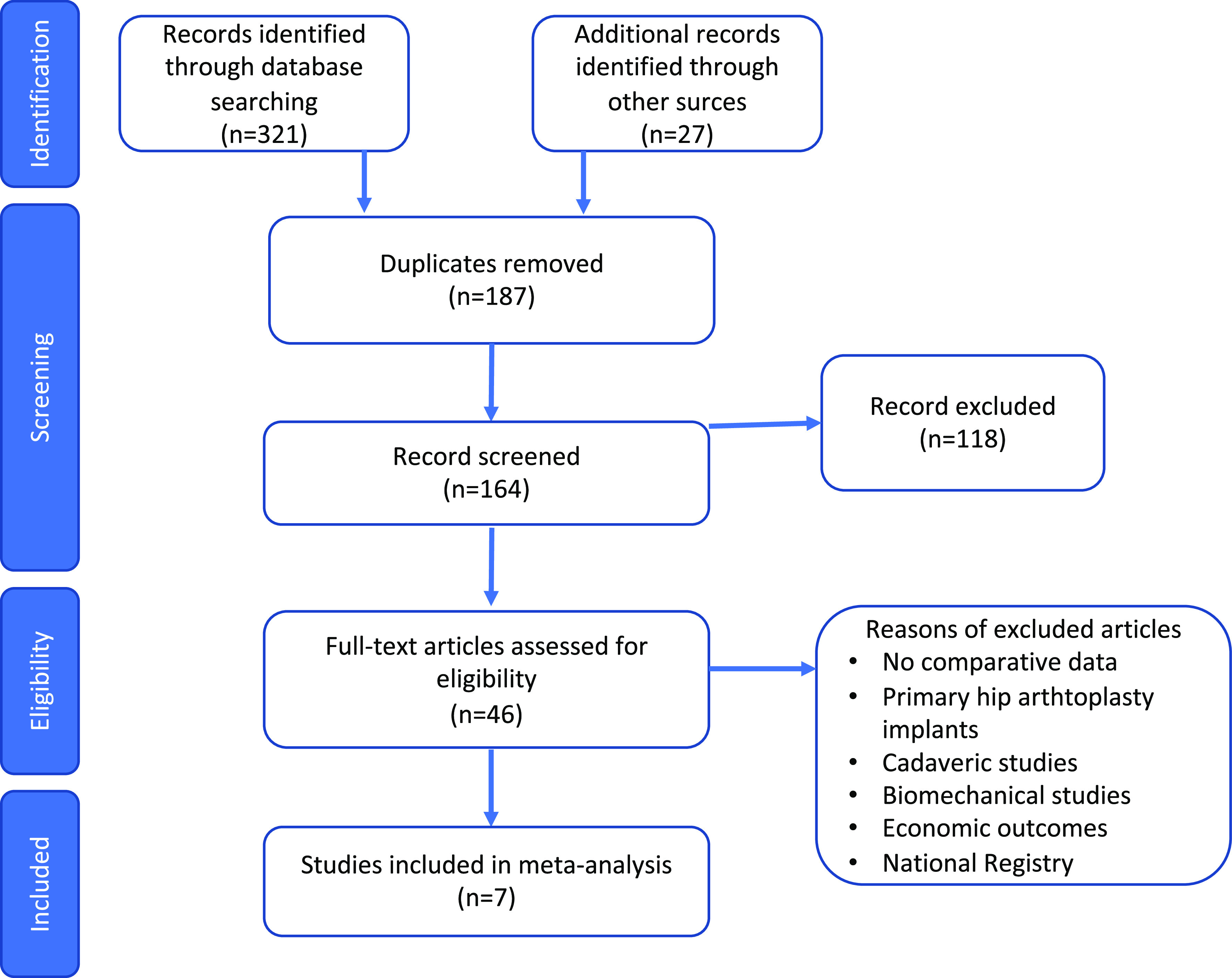
Quality of reporting of meta-analyses standards developed flow diagram.

All articles included in our study (Figure 1) had to satisfy the following inclusion criteria: studies of patients who underwent R-THA, including both DM or standard FB cup; separated results’ report of both DM and FB cup; the presence of outcome measurements’ report, with regards to:

- dislocation rates
- overall implant survivor;
- different manufacturers;
- use or non-use of bone cement;
- implants with different tribological properties.

Eligibility for inclusion criteria was accurately assessed by full and careful screening of all titles and abstracts. This was followed by the exclusion of studies not matching the set inclusion criteria and a full-text review of each included study. Furthermore, the Authors assessed and examined all articles’ bibliographies with the aim to look for additional sources. Bibliographies of previous systematic reviews were also examined.

Whereupon papers not meeting the aforementioned inclusion criteria were ruled out, this process excluding case reports or case series, review articles, expert opinions, and eventually studies not reporting dislocation rates. Final agreement and consensus between the two reviewers and the rest of the Authors were achieved.

After the final selection of studies meeting the inclusion criteria, the extrapolation of relevant key aspects of the included studies (the study design, number of study subjects with relative demographic data, time of publication) was performed. Extracted outcomes, where available, included also surgical approaches, Paprovsky classification, and duration of follow-up.

An institutional review board approval was not sought either acquired as all data were extracted from previously published studies. No external funding was received for the accomplishment of this study.

### Risk of bias

Previously published criteria were used to assess and classify the selected studies. This was done accordingly to the level of evidence (LOE) [13]. Two independent reviewers evaluated and assessed all studies’ methodology quality by using MINORS (methodological index for non-randomized studies) [14] in order to determine their potential bias in our protocol research. This is in keeping with the recommendations made by the Cochrane Observational Studies Methods Working Group [15].

A minimum follow-up of six months was considered an appropriate period of results monitoring accordingly to the evidence reported in the literature. In fact, it has been proved that the most common dislocations occur within the first six months after surgery (50–70%) [16].

A minimum global ideal score of 24 is usually necessary for a study to be considered high-quality. However, the included studies were judged as high quality (Table 1) if with a MINORS score between 20 and 24. This was decided as a higher event rate allows to give a more precise valuation of the influence of studied determinants. Therefore, we included the number of events in our risk of bias assessment.

**Table 1:** MINORS evaluation for selected studies.

	Clearly stated aim	Inclusion of consecutive patients	Prospective data collection	Endpoint appropriate to study aim	Unbiased assessment of study endpoint	Follow-up period appropriate to study aim	<5% lost to follow-up	Prospective calculation of study size	Adequate control group	Contemporary groups	Baseline equivalence of groups	Adequate statistical analyses	Total	Adequate number of patients	Risk of bias
Harwin et al. [20]	2	1	0	2	NA	1	0	0	2	1	1	2	11\24	Yes	High
Jauregui et al. [23]	2	2	0	2	NA	2	2	0	2	0	1	2	16\24	Yes	High
Stucinskas et al. [24]	2	2	0	1	NA	2	0	0	1	2	0	2	12\24	Yes	High
Schmidt et al. [19]	2	2	0	0	NA	1	2	0	1	2	1	2	13\24	Yes	High
Abdel et al. [21]	2	2	0	2	NA	1	0	1	2	2	1	1	14\24	Yes	High
Gonzalez et al. [22]	2	2	0	0	NA	2	0	2	2	2	2	2	16\24	Yes	High
Hernigou et al. [25]	2	0	0	2	NA	2	1	0	2	1	2	2	13\24	No	High
Assi et al. [26]	2	2	0	2	NA	1	2	0	2	2	1	2	16\24	No	High

## Statistical analysis

The meta-analysis was performed using RevMan V 5.2 (The Cochrane Collaboration, Copenhagen, Denmark) in order to calculate pooled summary and generate Forest plots. Continuous variables were extracted and analyzed as mean ± standard deviation (SD). If the SD was not expressed, a surrogate SD was calculated from the accessible data.

Relative risk (RR) with 95% CI was calculated for dislocation. Heterogeneity was tested using the χ^2^ and Higgins’ *I* ^2^ tests. A Mantel-Haenszel random-effect model was adopted if statistical heterogeneity was >50% at *I* ^2^ test; a fixed-effect model was used if statistical heterogeneity was below 50% [17, 18].

A subgroup analysis, evaluating the secondary outcome i.e. overall survivorship, infection, and aseptic loosening rates was conducted to gain more specific conclusions if the data were present. Forest plots were used to show the results of single studies.

Furthermore, publication bias was assessed using a funnel plot for all papers and for each previously quoted subgroup.

## Results

After carefully carrying out an exclusion procedure as explained, the eligible studies included for final consideration and analysis were only 8 [20–26]; out of those, none of the papers was classified as randomized controlled trial (RCT).

All articles were published between 2016 and 2019, describing the results of a total of 1777 R-THA (49.9% with DM acetabular cup, 50.1% with standard FB cup).

The sample size for each study ranged from 67 to 426 patients, with a mean age ranging from 57 to 73 years; most of the patients were women (53%). The mean follow-up period with regards to all finally included studies was ranged between 12 and 60 months. There was a statistically significant difference with regards to body mass index (BMI) reported in only one study [26], but this was considered an intrinsic feature of a specific study design.

The characteristics’ description of recruited articles and demographic data of patients enrolled in each paper is resumed in Table 2.

**Table 2:** Details of included studies.

Studies	Harwin et al. [20]	Jauregui et al. [23]	Stucinskas et al. [24]	Schmidt et al. [19]	Abdel [21]	Gonzalez et al. [22]	Hernigou et al. [25]	Assi et al. [26]
Min. follow-up (months)	48	30	24	12	24	52	60	74
Total operated hips	255	180	426	295	302	316	67	29
DM	85	60	247	184	126	150	35	16
FB	170	120	115	111	176	166	32	13
Mean age (years)	67	57	72	66	65	69	73	72.8
Female gender %	49%	57%	61%	54.9%	45%	51%	56.7%	77%
Total number of complications								
DM	5	3	11	24	11	7	1	0
FB	13	15	14	19	34	21	7	3
Infections								
DM	1	1	5	10	3	4	NR	0
FB	2	3	2	4	9	4	NR	0
Dislocations								
DM	1	1	5	2	4	1	1	3
FB	6	7	10	10	17	7	7	0
Intraprostetic dislocations	0	0	1	0	NR	0	0	NR
Aseptic loosening								
DM	2	1	1	3	4	1	0	0
FB	1	5	1	4	8	8	0	0
End of follow-up survivorship								
DM	81	57	235	271	115	143	32	16
FB	161	108	100	165	142	145	27	10

The surgical approach performed and Paprovsky classification was not always reported in the included studies.

The quality of selected studies was evaluated as “moderate” if found with a mean MINORS score of 13.5 points (range 11–16). None of the papers got a good global score (>20); a low risk of selection bias was attributed to studies with a minimum postoperative follow-up greater than 12 months, but all included studies were reported to have a retrospective design. A blind evaluation of objective endpoints with regards to reports of surgical procedures cannot be performed because of the intrinsic nature of the topic discussed. This should be considered as an inevitable source of potentially high-performance bias. Despite the fact that contemporaneity at the time of surgery was not always respected, the control group was found to be reasonable in all cases.

**Figure 2:**
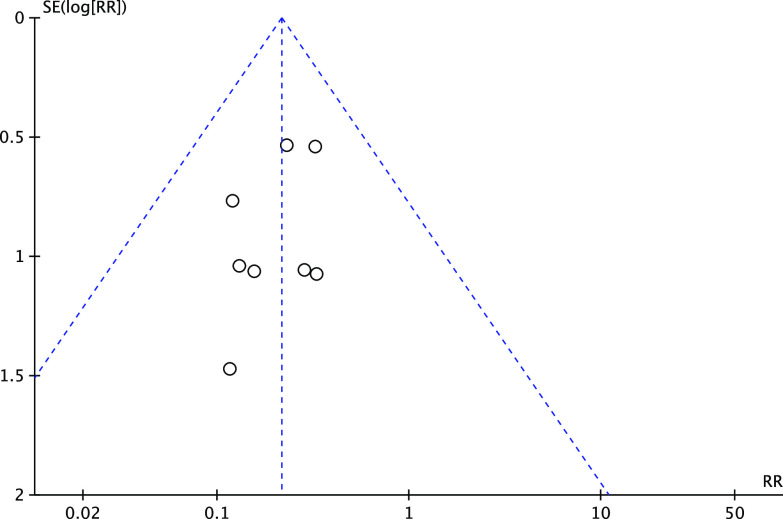
Funnel plot for all studies included.

Funnel plots (Figure 2 for all papers included did not show any asymmetry, excluding any possible publication bias.

**Figure 3:**
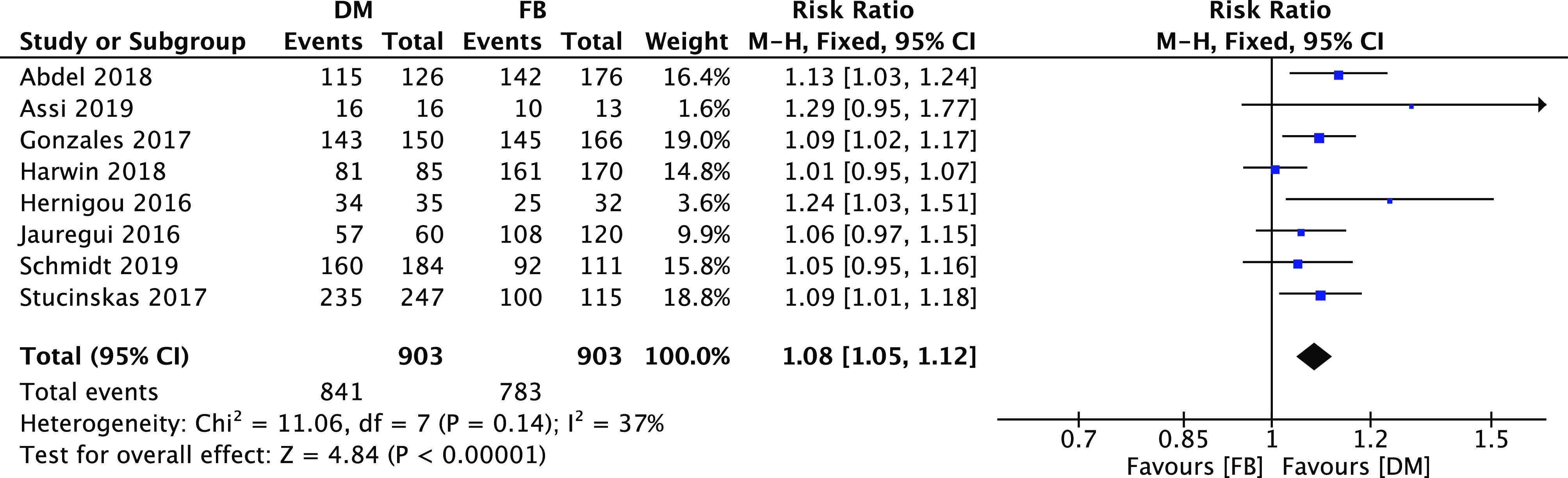
Forest plot for overall survivorship DM vs. FB.

The details of the selected studies included in the meta-analysis yielded the following results (Figure 3) regarding the overall implant survival: these presented a slight significant risk ratio of 1.08 [1.05, 1.12] (95% CI, *I* ^2^=37%, *P*<0.00001) with a statistically significant difference between the two studied groups in favor of the DM implant.

**Figure 4:**
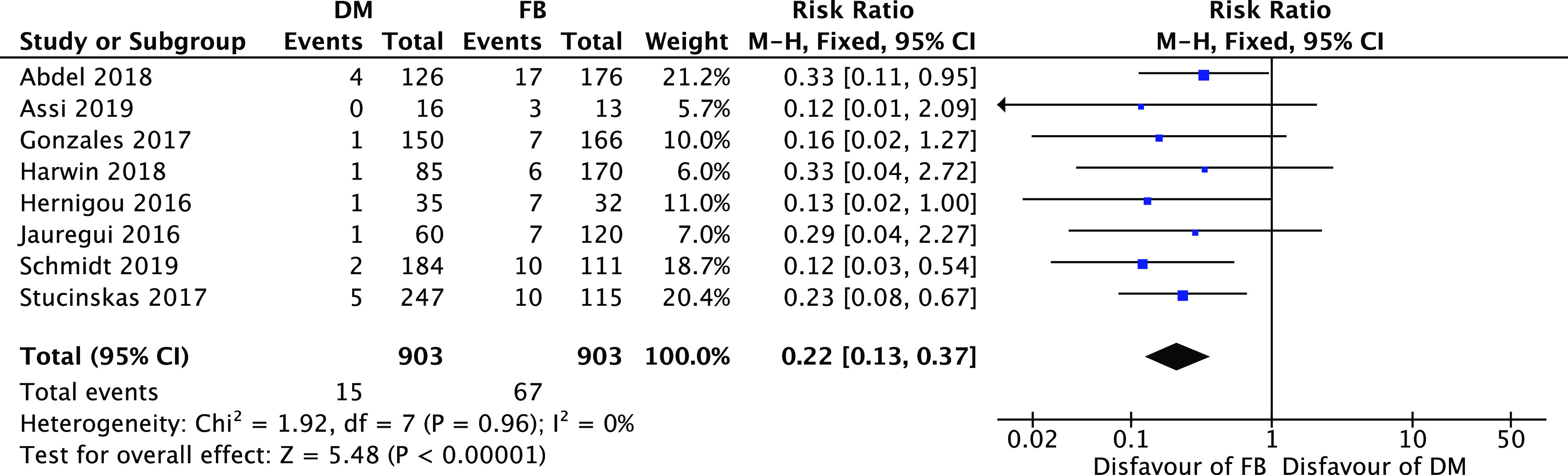
Forest plot for dislocation rate.

**Figure 5:**
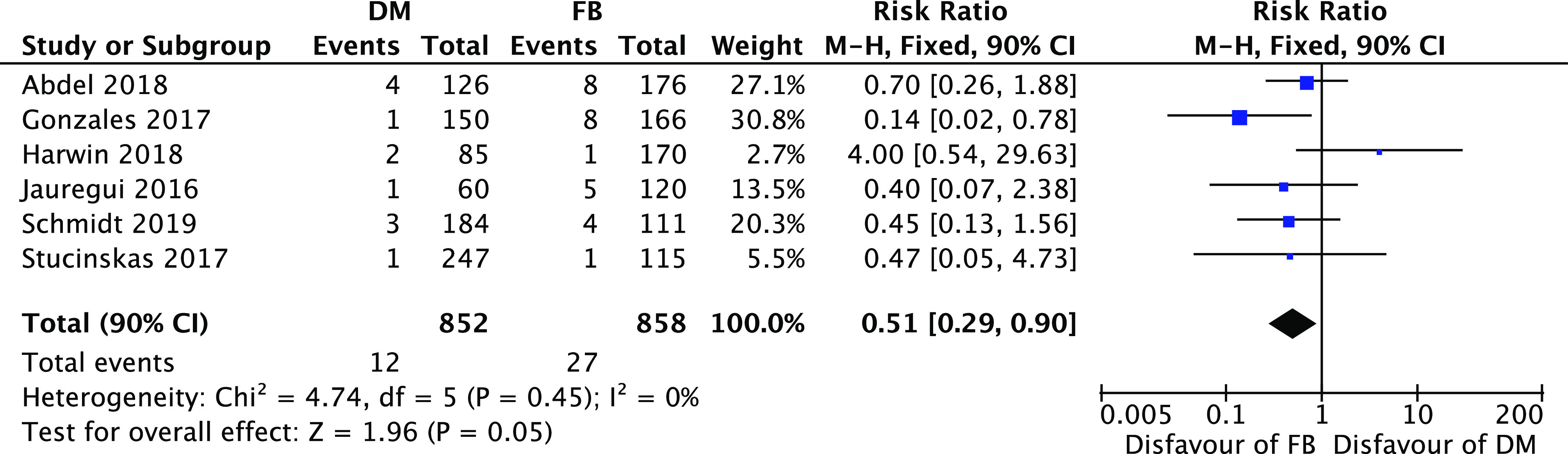
Forest plot for aseptic loosening.

A statistically significant difference (Figure 4) in favor of the DM group was recorded considering only the dislocation rate risk ratio 0.22 [0.13, 0.37] (95% CI, *I* ^2^=0%; *P*<0.00001) and the aseptic loosening risk 0.51 [0.29, 0.90] (95% CI, *I* ^2^=0%; *P*<0.05) as well (Figure 5).

**Figure 6:**
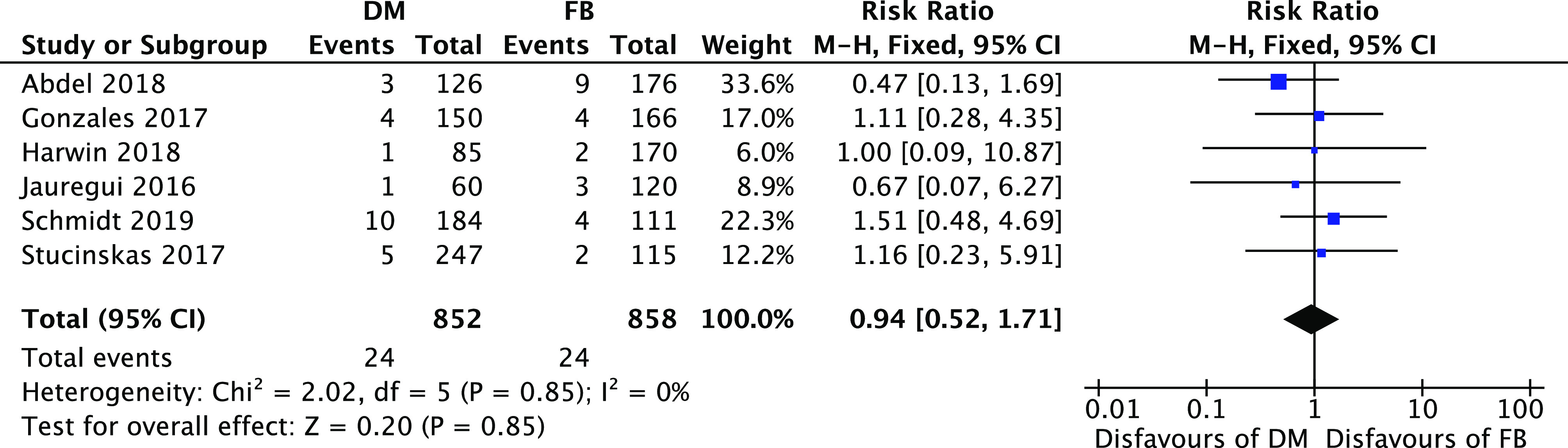
Forest plot for infection risk.

No statistical differences were found between the two group with regards to the risk ratio of infection 0.94 [0.52, 1.71] (95% CI, *I* ^2^=0%; *P*=0.85) (Figure 6).

## Discussion

Gilles Bousquet and André Rambert initially brought the concept of a design characterized by two distinct articulations in THA (France, 1974) [27], as an attempt to diminish the risk of dislocation. This technology achieved widespread success over the following years, thought to be based on the presence of a larger effective head diameter, which was found to allow a greater range of motion before complete dislocation, and consequently increase the stability of the implant [28, 29].

The main finding arising from the data collected, analyzed, and presented in our study is that DM for R-THA is more effective compared to standard FB cups in terms of implant survivor and prevention of dislocation at short and medium follow-up. This is true as attested by the most up-to-date available scientific evidence. However, we should take into consideration the scarceness and the relatively low methodological quality of the studies included in our review and that support our statistical analysis. Despite this weakness, a lower rate of dislocation in the DM group was strongly highlighted in all included works; do note a statistically significant difference between the two studied groups was not reported due to the small number of cases examined.

It has been reported that one of the advantages of the use of DMC is a decreased rate of THA and R-THA dislocation. The Kaiser-Permanente Register reports instability as one of the commonest complications and the greatest indication for R-THA (49.8% of the total) [30]. Similar data and considerations have been similarly reported in the United States National Inpatients Sample Database [31], the National Joint Replacement Register in Australia [32], and the Swedish Hip Arthroplasty Register [33]. These reports highlight a revision rate for instability being between 14.6% and 22.5% for first revision surgery, and 25.6% and 31.1% for re-revision surgery. It seems that implant dislocations more commonly occur during the first 2 post-op years [34]. However, the overall dislocation rate has been variably reported as being between 14% and 21% [35, 36].

In contrast with what has been shown by works on surgical treatment options for R-THA, the DM cup was found to provide a low risk of dislocation after R-THA, as documented in several study reports. However, very few studies report data on comparison between DM and FB in the same cohort. This aspect does not allow a full objective comparison between the two groups.

Vasukutty et al. [37] reported in their work a dislocation rate being between 2% at 42 months of follow-up when showing results of 155 R-THAs treated with DM. Philippot et al. [38] found comparable results with a 3.7% of dislocation rate (163 R-THAs) using DM at 60 months of follow-up. Leiber-Wackenheim et al. reported a post-op recurrent dislocation rate of 1.7% after acetabular revisions for recurrent dislocation using cementless DM. An odds ratio for the DM group of 0.253 (p 0.1) was reported by Leiber-Wackenheim et al., with the interesting implication in favor of the DM group of providing a protector effect in preventing dislocation [39].

Similar results were however recorded in further studies. These were again in favor of DMC and showed a protective effect from dislocation within 6 months of follow-up [40]. Ozden et al. highlighted that DM could be able to provide excellent stability with no cases of dislocation and an excellent survival rate of 93% at 5 years, even in cases with abductor-trochanteric complex deficiency [41].

An increasing amount of evidence is providing support in favor of the efficiency of using DM cup systems in R-THA with low dislocation rates [42–47] but currently, there is no study in the literature that demonstrates this with statistically significant evidence. Various studies have reported that DM cups in revision surgery can be associated with a reduced risk of dislocation. Hailer et al. [44] found the risk of pre-revision after DM cup insertion for revision THA performed due to recurrent dislocations appears low.

The demonstrated superiority of DM is further confirmed when considering results from works in which the DM option was used in patients considered at high risk of complications (extensive acetabular deficiencies, important muscle defeats, neuromuscular or cognitive disorders) [48–50].

The aforementioned findings are in keeping with those published in the international literature, with regards to the ones obtained from single-center reports, revisions, and national registry data, either for THA and R-THA.

Regardless of these encouraging results and the widespread increased use of this technology, issues related to the additional bearing surface have been raised. In fact, this aspect is thought to be able to increase the risk of infection and the possibility of accelerated wear. Therefore, the use of these components can be considered to cause a consequent growth in the risk of aseptic loosening [51].

However, this has not been demonstrated yet with a high level of evidence and further studies are definitely needed in order to validate or refute these hypotheses [51].

A remarkable low dislocation rate at short and medium follow-up (with a range from 0 to 1.5%) has been reported by few Authors when choosing the DM option [24, 52]. However we must stress that the relatively low number of patients included in the majority of these studies unequivocally affected the strength of such good results.

Although the presence of very good results in terms of the total volume of wear when using conventional metal-PE bearings with 22.2mm femoral heads [53] and the contemporary presence in the literature of encouraging results at medium follow-up with regards to newly introduced highly cross-linked PE were recently reported [54]. The main weakness related to such results is that benefits at long follow-up terms have not been yet demonstrated but only hypothesized. Given that late dislocation rate after five years is often related to PE wear, conclusive findings cannot yet be inferred and room for uncertainty still remains, also regarding the balance between pros and cons of different designs.

The possibility of intra-prosthetic dislocation (IPD) occurrence has been considered as a serious potential limitation of DM implants. This is a specific type of failure of these designs reported primarily for the first generation of implants. Aspects related to the wear seem to be essential parts of the main mechanisms of failure of these types of implants. Moreover, recent studies on newer generation DM implants have produced reports of early IPD. Therefore, further high levels of evidence research with a longer period of surveillance are strictly required in order to clarify this essential aspect [9].

Given the lower dislocation-rate and re-revisions for dislocations at midterm, dual-mobility constructs should not be chosen to compensate for poor surgical technique or technical errors [50].

In our systematic review, we found only one case of IPD [24]. However, we must state that IPD was seen in the early years of DM use, then its occurrence nowadays became exceptional.

This review shows several limitations. Firstly, a retrospective study design is adopted in all included studies which makes the studies qualitatively of low methodology. Practically the level of evidence of our review is limited by these aforementioned aspects. However, no evidence of publication bias has been reported as yet. This point is highlighted in the funnel plot. Secondly, we must report extreme heterogeneity with regards to diagnosis, demographics, and indications for surgery of the patients included in the scrutinized studies; however, for such niche surgery finding heterogeneous groups is thought to be very rare and complicated, if not impossible. Anyhow, we found a statistically significant difference in the two cohorts but we considered this as an intrinsic aspect of most papers’ study design. This evidence supports and justifies the use of DM technology for R-THA, particularly in higher-risk patients, against the use of other implant designs. We interpreted this as being a strength in support of our statistical analysis and results.

The follow-up period of the papers included in our review is too brief to clarify and assure whether the efficacy of the DM option in reducing the dislocation rate can be corroborated at longer follow-up terms. Despite the mentioned weaknesses, this evidence can be considered sufficiently significant given the fact that most of the episodes of DM implant dislocations happen in the first six months after surgery, and this has already and widely been reported in the literature with a very high level of evidence.

## Conclusion

Our Meta-analysis provides evidence of the advantages of DM cup against FB cup for R-THA. The use of a DM cup for R-THA (independently of indications) seems to be able to decrease the risk of implant failure at mid-term follow-up, reducing at the same time early post-op dislocation rates and THA re-revision rates when comparing to results obtained with FB cups. There is no significant level of evidence that the use of DM increases the risk of infection compared with FB. FB implants have a higher risk rate of aseptic loosening at mid-term follow-up.

Further prospective studies with a high level of evidence are needed in order to validate the reported temporary results and shed a light on the uncertain aspects.

## Conflict of interest

Authors had nothing to disclose about conflict of interest.
